# Long-Term Efficacy of Intensive Zoledronate Therapy and Predictors of Retreatment in Paget’s Disease of Bone

**DOI:** 10.1007/s00223-021-00848-x

**Published:** 2021-04-19

**Authors:** Marco Barale, Sarah Sigrist, Fabio Bioletto, Federica Maiorino, Ezio Ghigo, Riccardo Mazzetti, Massimo Procopio

**Affiliations:** 1 Division of Endocrinology, Department of General and Specialty Medicine, Diabetology and Metabolic Diseases, Molinette Hospital, University of Turin, Cso Dogliotti, 14, 10126 Turin, Italy; 2 Division of Endocrinology, Diabetology, Osteology and Metabolic Diseases, Kantonsspital St.Gallen, Rorschacher Strasse, 95, 9007 St.Gallen, CH Switzerland; 3 Radiology Unit, Department of Diagnostic Imaging and Interventional Radiology, Molinette Hospital, University of Turin, Cso Dogliotti, 14, 10126 Turin, Italy

**Keywords:** Paget’s disease of bone, Zoledronate, Intensive therapy, Bone alkaline phosphatase

## Abstract

Despite the current debate on the best therapeutic approach, i.e. symptomatic vs intensive strategy, one zoledronate (Zol) infusion is effective in most patients with Paget’s disease of bone (PDB), whereas few need retreatment, whose predictors are not well established. We aimed to evaluate long-term efficacy of intensive Zol therapy and predictors of retreatment in PDB. Pagetic complications, clinical and biochemical response to Zol together with frequency of retreatment were retrospectively assessed in forty-seven PDB patients (age, mean ± SD: 72.5 ± 8.9 years, M/F: 24/23; symptomatic/asymptomatic: 16/31). Statistical analysis for retreatment prediction were based on Mann–Whitney *U* test, Pearson’s Χ2 and ROC curve analysis. During seven-year follow-up, all patients achieved pain relief and only one underwent arthroplasty. Bone alkaline phosphatase (BAP) detected three non-responder (6%) and six relapsing (13%) patients needing retreatment. Retreated patients had less old age (66.1 ± 11.2 vs 74.0 ± 7.7 years), higher frequency of polyostotic disease (78% vs 40%) and higher baseline (96.5 ± 24.8 vs 44.9 ± 27.7 mcg/l) and post-Zol nadir BAP levels (24.7 ± 24.1 vs 8.1 ± 4.1 mcg/l) than patients treated once (*p* < 0.05 for all comparisons). In multivariate analysis both serum baseline and post-Zol nadir BAP significantly predicted retreatment (OR 1.09, 95%CI 1.01–1.17 and 1.29, 1.03–1.62, respectively), with ROC curve analysis showing the greatest accuracies for threshold values of 75.6 and 9.9 mcg/l (sensitivity 88 and 90%, specificity 94 and 86%, AUC 0.92 and 0.93, respectively). Our data in mostly asymptomatic, metabolically active PDB patients treated with intensive Zol therapy show a negligible incidence of pagetic complications and long-term optimal disease control, with BAP being the best predictor of retreatment.

## Introduction

Paget’s disease of bone (PDB) is a chronic focal disorder of bone remodelling, affecting one (monostotic, MO-PDB) or more skeletal sites (polyostotic, PO-PDB), and characterized by increased bone resorption followed by imperfect osteoblast-mediated bone repair [1, 2]. Both genetic and environmental factors seem to play a pathogenetic role in development of PDB, although the etiology remains uncertain [3–5]. PDB is most frequently asymptomatic and often incidentally discovered while evaluating total plasma alkaline phosphatase (ALP) or performing radiographs for other purposes. The most common clinical manifestation is pain at focal involved site(s), while long-term complications, connoting natural history of PDB, include fragility fracture, osteoarthritis, spinal stenosis, bone deformity, deafness, high-output cardiac failure, hypercalcaemia and cardio-metabolic disorders. Moreover, neoplastic degeneration in osteosarcoma or giant cell tumor are rarely observed [6, 7]. Elevation of serum ALP is an important clue to the diagnosis of PDB, which is based on the detection of typical radiological features on plain radiographs of involved site(s). If ALP values are normal but the clinical suspicion of metabolically active PDB is high or when an abnormal liver or biliary tract function occurs, measurement of more specific bone turnover markers such as bone ALP (BAP), procollagen type I N-terminal propeptide (PINP) or urinary cross-linked N-terminal telopeptide of type I collagen (uNTX) is indicated [8]. In addition, radionuclide bone scan is recommended for the assessment of disease extent and bone metabolic activity [9].

Nowadays, it is debated on the best therapeutic approach to PDB patients. In fact, bisphosphonate treatment, with zoledronate being the first-line agent, is advised in most patients with active disease who are at risk for pagetic complications, according to what is known as intensive therapeutic strategy [9, 10]. However, recent guidelines on PDB recommend treatment only for pain relief [11], lacking the evidence of its benefit in preventing disease complications in asymptomatic patients [12–14]. Therefore, long-term observational studies in these PDB patients could be informative about the incidence of pagetic sequelae. Moreover, some patients do not respond to a single infusion of zoledronate or they achieve a transient disease control with the need of retreatment [15–19], whose predictors are not well established [15, 20]. Particularly, it remains to be clarified whether the extent of PDB or other clinical characteristics as well as serum BAP monitoring could play a role in forecasting disease response and long-term risk of relapse after zoledronate treatment.

Aim of our retrospective long-term study was to assess the clinical benefit, the incidence of disease complications and the rate of bone turnover reduction in mostly asymptomatic metabolically active PDB patients undergoing intensive zoledronate treatment. Moreover, we looked for clinical and biochemical factors associated to zoledronate retreatment in non-responder and relapsing PDB patients after the first infusion.

## Materials and Methods

### Study Setting and Participants

We performed a retrospective review of the medical charts of all patients with PDB (*n* = 54) referred to our Bone Metabolism Diseases Center and treated with intravenous zoledronate from January 2004 until December 2018. A 4-mg dose (Zometa®) was used as off-label therapy up to May 2008 whereas, thereafter, a 5-mg dose (Aclasta®), another brand registered for PDB, was administered. Fifteen patients with missing pre- and/or post-treatment data were contacted and invited for a follow-up visit to complete medical information and biochemical data. Untraceable patients (*n* = 5) and those affected by interfering conditions (primary hyperparathyroidism, *n* = 1; breast cancer, *n* = 1) were excluded from the study. Patients with PDB were diagnosed and treated in line with the classical intensive approach and in accordance with clinical guidelines on the management of PDB [9, 21].

### Study Measurements and Outcomes

The diagnosis of PDB was based on the detection of typical focal bone lesions on plain radiographs, while bone metabolic activity was revealed by serum ALP and BAP levels. The disease extent evaluation was based on bone scintigraphy, which allowed us to identify patients with MO- and PO-PDB. Specifically, at baseline, in 47 PDB patients included in this study, a detailed clinical history comprising clinical manifestations, disease duration, previous specific therapy for PDB, concomitant treatments with calcium and vitamin D was collected. Moreover, a comprehensive biochemical panel, specifically ALP, BAP, creatinine, parathormone, 25 (OH) vitaminD, serum total and ionized as well as urinary calcium levels were evaluated. The same bone turnover markers, serum creatinine, 25 (OH) vitaminD, total and urinary calcium levels were available during a long-term follow-up after infusion of intravenous zoledronate. In addition, clinical complaints were registered at each follow-up visit.

We defined therapeutic response at six months as the normalization of ALP or a reduction of at least 75 percent in the ALP excess (the difference from the midpoint of the reference range), as already reported by other Authors [22]. In addition, we adopted the same criteria for the definition of therapeutic response using BAP changes. Moreover, the relapse of PDB was defined as a rise of ALP or BAP levels above the reference range after therapeutic response [23]. For responding patients whose ALP or BAP levels remained above the normal range, disease relapse was defined as an increase above response levels at six months. Zoledronate retreatment was performed in non-responder patients, who did not reach target BAP levels, and immediately after disease relapse defined according to BAP.

ALP (UI/l) was tested using colorimetric assay in accordance with a standardized method (Cobas, Roche). Serum BAP (µg/l) was measured by an immunoradiometric sandwich method that use mouse monoclonal antibodies directed against two different epitopes of BAP and hence not competing (Beckman Coulter). Serum total and urinary calcium (mmol/l and mmol/die) were tested using automated methods based on colorimetric and enzymatic assays (Cobas, Roche). For serum ionized calcium (mmol/l) a specific probe was used after correction for pH. Intact PTH assay (pmol/l) based on an immunoradiometric sandwich method (IRMA) that used two polyclonal antibodies (DiaSorin). Serum 25 (OH) vitaminD (nmol/l) was tested by a radioimmunoassay method using an antibody with specificity to 25 (OH) vitaminD (DiaSorin). Plasma creatinine levels (µmol/l), was measured by enzymatic colorimetric tests (Cobas, Roche). Glomerular filtration rate (GFR, ml/s) was calculated according to Cockroft-Gault formula.

Plain radiographs of all bone lesions were obtained before zoledronate administration and interpreted by an experienced radiologist, while bone scans were acquired 3 h after injection of 99mTc-labelled methilen diphosphonate, using a large field-of-view dual-detector camera.

### Statistical Methods

Data are presented as mean ± SD or as mean, 95% confidence interval (CI). Normality of frequency distribution functions was tested by the Shapiro–Wilk *W* test. Significant differences were sought by the Mann–Whitney *U* test, ANOVA test or Pearson’s Χ2 analysis. ROC curve analysis was used to determine an optimal threshold value of baseline and nadir ALP and BAP to predict the need of retreatment. Logistic regression analysis was used to assess the relationship of rate of retreatment with clinical and biochemical factors; data are presented as odds ratios (OR) and 95%CI, while Nagelkerke R2 expressed the amount of variance of dependent variable due to independent variables. Calculations were performed using SPSS Windows release 24.0; *p* < 0.05 was considered significant.

## Results

### Baseline Assessment

The characterization of all forty-seven patients with PDB, stratified as MO-PDB (53%) and PO-PDB (47%) is reported in Table 1. 18 out of 47 PDB (i.e. 38%) patients had not been previously responsive to oral bisphosphonates and nobody had been undergone intravenous bisphosphonates. All patients were on calcium carbonate and cholecalciferol supplementation, specifically with mean dosages equal to 457 ± 480 mg/die and 832 ± 261 UI/die, respectively. As reported, PO-PDB and MO-PDB differed only in baseline BAP, whose levels were higher in the former than in the latter. The most frequently affected bones were the pelvis (81%), vertebrae (43%), femur (19%) and skull (15%), whereas other localizations included knee, tibia, ankle, foot, sternum and shoulder. Ten out of 22 patients with PO-PDB showed 3 or more sites affected by pagetic lesions. Baseline characteristics of excluded patients (*n* = 7) were similar to the studied group (data not shown).

**Table 1:** Baseline clinical and biochemical features in patients with Paget’s disease of bone (PDB) as a whole or separately as patients with polyostotic (PO-PDB) and monostotic (MO-PDB) disease

	PDB (*n* = 47)	PO-PDB (*n* = 22)	MO-PDB (*n* = 25)	Ref. range
Sex (M / F)	24/23	11/11	13/12	
Age (years)	72.5 ± 8.9	71.5 ± 10.5	73.4 ± 7.4	
BMI (kg/m^2^)	27.2 ± 5.5	26.1 ± 5.8	28.1 ± 5.3	18.5–24.9
Time since PDB diagnosis (years)	6.9 ± 8.6	6.4 ± 9.3	7.3 ± 8.2	
ALP (UI/l)	449.0 ± 682.9	608.8 ± 908.4	272.4 ± 175.7	50–150
BAP (mcg/l)	53.3 ± 33.1	65.5 ± 34.9^a^	41.7 ± 27.3	< 21
Serum total calcium (mmol/l)	2.3 ± 0.1	2.3 ± 0.2	2.3 ± 0.1	2.2–2.6
Serum ionized calcium (mmol/l)	1.2 ± 0.1	1.1 ± 0.1	1.2 ± 0.1	1.1–1.3
Urinary calcium (mmol/die)	2.7 ± 1.5	2.9 ± 0.8	2.6 ± 1.8	2.5–7.5
Parathormone (pmol/l)	4.85 ± 2.90	4.33 ± 1.47	5.33 ± 3.79	1.05–6.90
25(OH) vitamin D (nmol/l)	54.7 ± 25.5	60.9 ± 24.5	48.7 ± 25.7	75–125
Serum creatinine (µmol/l)	75.4 ± 16.0	76.5 ± 19.5	74.5 ± 12.7	44–106
Creatinine clearance (ml/s)	1.3 ± 0.4	1.2 ± 0.4	1.4 ± 0.2	1.2–2.0
Bone pain at involved pagetic site(s)	16 (34)	9 (41)	7 (28)	
Pagetic fractures^b^	5 (11)	3 (14)	2 (8)	
Deafness	1 (2)	1 (5)	0 (0)	
Bone deformities	3 (6)	2 (9)	1 (4)	
Osteoarthritis	18 (38)	10 (45)	8 (32)	

Data are presented as mean ± standard deviation or n (percentage). Statistical analysis is performed by Mann–Whitney test and Pearson’s Χ2

*BMI* body mass index, *ALP* alkaline phosphatase, *BAP* bone alkaline phosphatase

^a^*p* < 0.05 vs MO-PDB

^b^Specifically, pagetic fractures occurred at vertebral sites (*n* = 4) and at the hip (*n* = 1)

### Follow-Up: Disease Control and Zoledronate Retreatment

Out of 47 patients, 11 were treated with Zometa® (zoledronate 4 mg IV) and 36 with Aclasta® (zoledronate 5 mg IV) with a follow-up of 84 months. Zoledronate infusions were generally well-tolerated. The most frequent registered side-effects were transient fever and musculoskeletal pain, which affected 11% of treated patients.

**Fig. 1:**
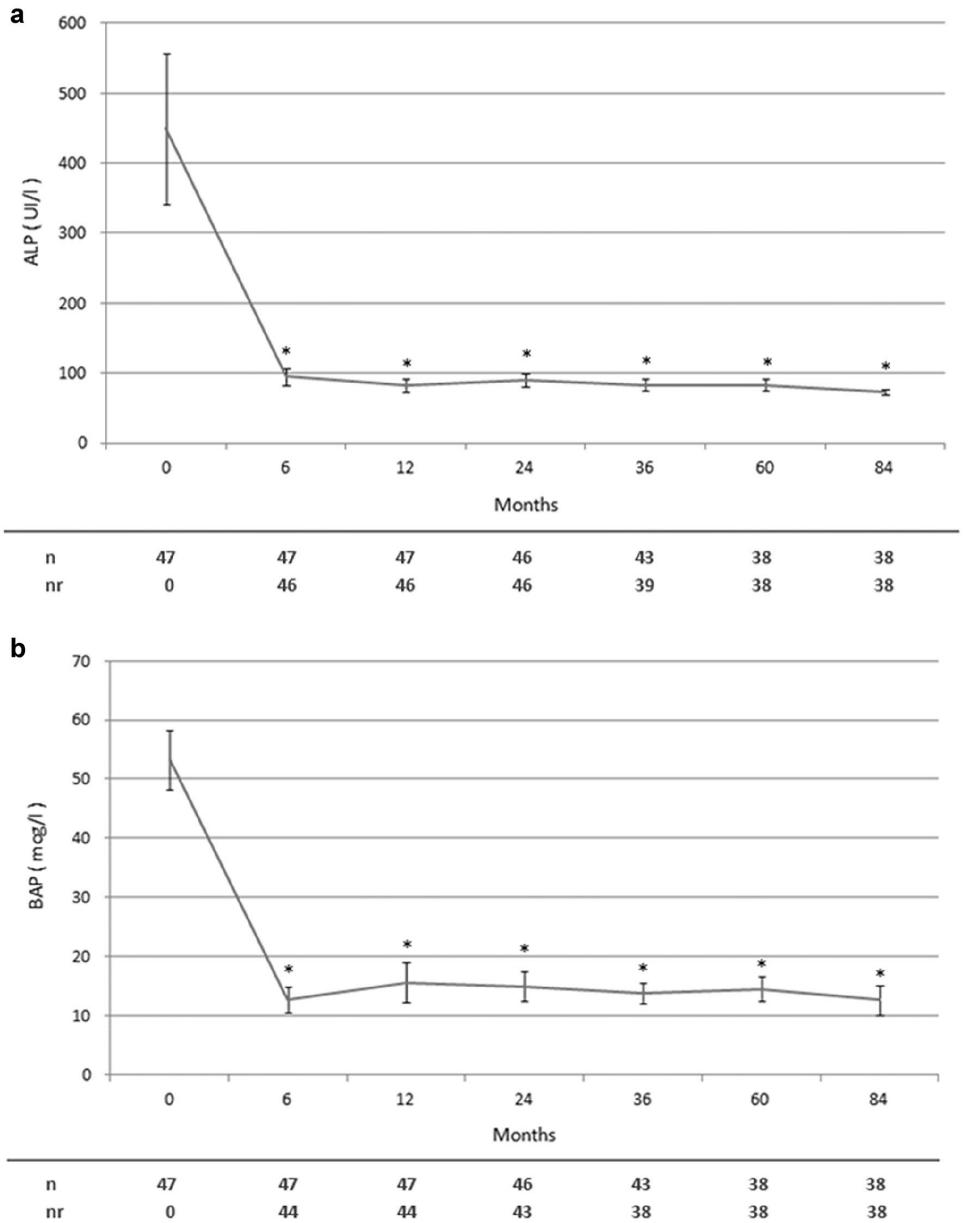
Serum total (ALP, **a**) and bone (BAP, **b**) alkaline phosphatase levels over time in all patients with Paget’s disease of bone before and after the first infusion of zoledronate. Patients repeating treatment were subsequently excluded from the analysis. Data are represented as mean ± standard error of the mean. *p < 0.05 vs basal values. *n* number of patients assessed at each time point, *nr* number of patients with ALP or BAP within reference range at each time point

Clinical and biochemical data during the follow-up were available at six and twelve months since the first zoledronate course. Subsequently they were registered every 12 (± 4) until 36 months and then every 24 (± 8) until 84 months. All symptomatic patients (34%) had amelioration of bone pain, persisting during the entire follow-up, though it could not be statistically evaluated because a quantitative bone pain assessment was not routinely performed. Considering disease complications, only one patient underwent arthroplasty due to knee osteoarthritis. On the other hand, serum ALP and BAP response after zoledronate infusion as well as their levels during the entire follow up are shown in Table 2 and Fig. 1, respectively. As reported, in comparison to baseline, the reduction of both ALP and BAP values was statistically significant during the whole period. Overall, most patients undergoing a single infusion of zoledronate achieved and maintained a good long-term disease control (*n* = 38). Renal function, 25 (OH) vitaminD, serum total and urinary calcium levels did not significantly change over time after the first infusion of zoledronate (data not shown).

**Table 2:** Serum ALP and BAP response to zoledronate infusion and need of retreatment in all patients with Paget’s disease of bone (PDB) and in those with polyostotic (PO-PDB) and monostotic (MO-PDB) disease

	PDB (*n* = 47)	PO-PDB (*n* = 22)	MO-PDB (*n* = 25)
Therapeutic response according to ALP	46 (98)	21 (95)	25 (100)
Therapeutic response according to BAP	44 (94)	19 (86)^a^	25 (100)
Relapse according to ALP	4 (9)	3 (14)	1 (4)
Relapse according to BAP	6 (13)	4 (18)	2 (8)
Retreated patients	9 (19)	7 (32)^a^	2 (8)
ALP Nadir (UI/l)	68.8 ± 21.8	68.9 ± 18.9	68.5 ± 27.4
BAP Nadir (mcg/l)	8.6 ± 4.0	10.0 ± 4.3^a^	6.3 ± 2.3
Time to ALP nadir (months)	18.2 ± 11.3	18.7 ± 12.8	17.4 ± 9.1
Time to BAP nadir (months)	17.4 ± 10.9	21.3 ± 11.6^a^	11.0 ± 5.6

Data are presented as n (percentage) or mean ± standard deviation. Statistical analysis is performed by Pearson’s Χ2 and Mann–Whitney test

^a^*p* < 0.05 vs MO-PDB

Moreover, the relapse of PDB occurred in 9% (*n* = 4) and 13% (*n* = 6) of patients, considering a rise of ALP or BAP levels, respectively, and it was registered after a mean of 33 ± 6 and 34 ± 5 months since the first infusion. When considering patients previously treated with oral bisphosphonates, we found no differences in ALP or BAP therapeutic response rate and frequency of relapse in comparison to untreated patients (data not shown). Moreover, as expected, the former had a longer disease duration compared to the latter (10.9 ± 9.7 vs 3.4 ± 6.7 years, *p* < 0.05).

**Fig. 2:**
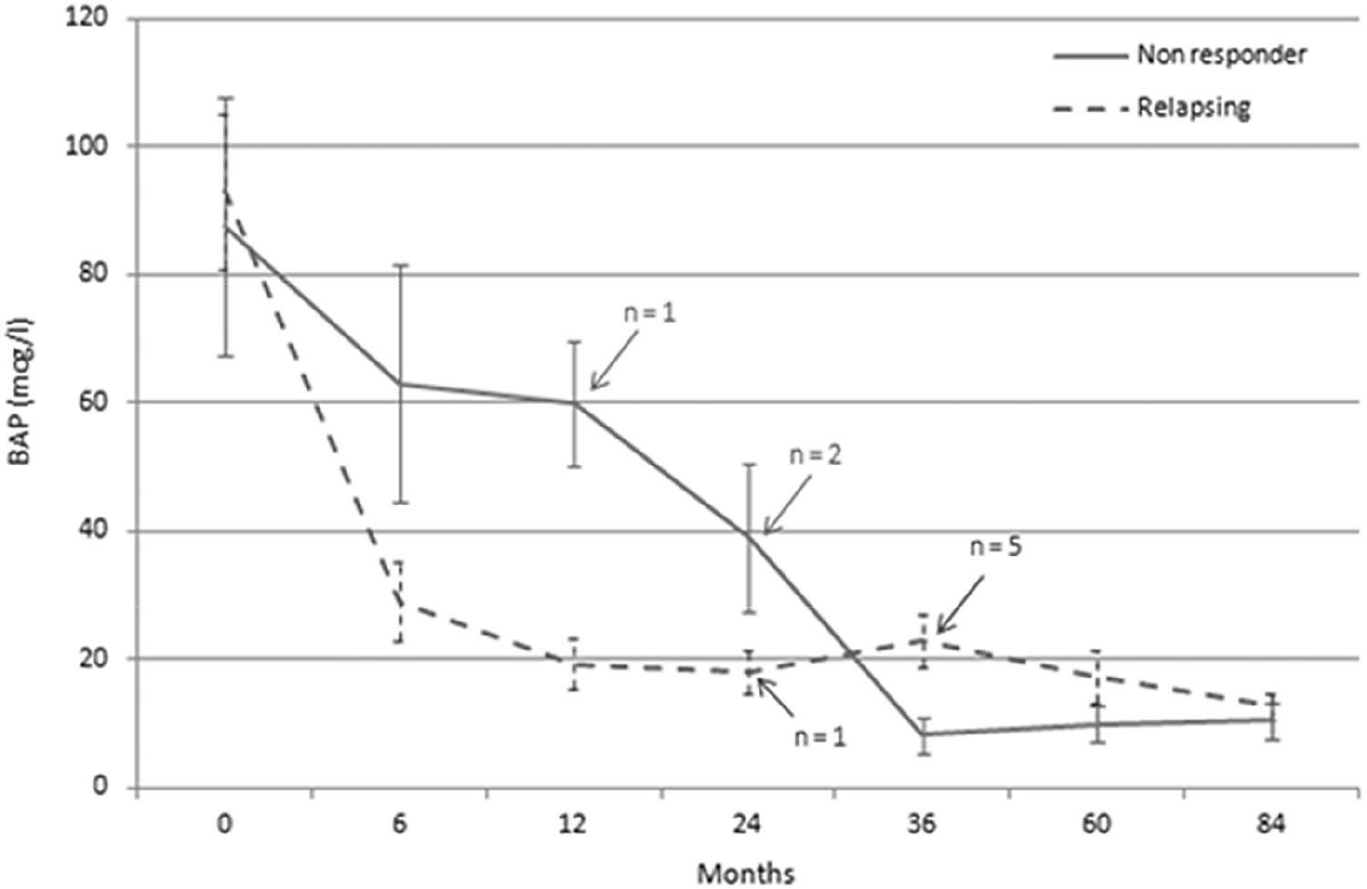
Serum bone alkaline phosphatase (BAP) levels over time in non-responder patients and in those who experienced biochemical relapse after the first infusion of zoledronate. Data are represented as mean ± standard error of the mean. The arrows indicate the number of patients retreated after each time point

Non-responding and relapsing patients, whose serum BAP levels over time are showed in Fig. 2, underwent one more zoledronate infusion after a mean of 2.4 ± 0.7 years since the first one. As shown in Table 3, patients needing retreatment were less old and they had higher basal and post-zoledronate nadir levels of both ALP and BAP, respectively, than those who had a good disease control. Moreover, retreated patients had a higher prevalence of PO-PDB, spine localizations and prevalent pagetic fractures in comparison to single-treated patients.

**Table 3:** Baseline clinical and biochemical features as well as total (ALP) and bone alkaline phosphatase (BAP) nadir levels after zoledronate treatment in patients with Paget’s disease of bone (PDB) needing retreatment (R-PDB) and achieving a good disease control with a single infusion (C-PDB)

	R-PDB (*n* = 9)	C-PDB (*n* = 38)	Ref. range
Sex (M / F)	5 / 4	19 / 19	
Age (years)	66.1 ± 11.2^a^	74.0 ± 7.7	
BMI (kg/m^2^)	30.9 ± 9.3	26.5 ± 4.3	18.5–24.9
Time since PDB diagnosis (years)	11.8 ± 12.4	5.6 ± 7.1	
Poliostotic disease	7 (78)^a^	15 (40)	
Pelvis pagetic localization	7 (78)	31 (82)	
Spine pagetic localization	7 (78)^a^	13 (34)	
Femur pagetic localization	2 (22)	7 (18)	
Skull pagetic localization	1 (11)	6 (16)	
Pagetic fractures	3 (33)^a^	2 (5)	
N° of pts treated with Zol (4/5 mg)	2/7	9 / 29	
ALP (UI/l)	913.6 ± 685.8^a^	350.5 ± 650.4	50–150
BAP (mcg/l)	96.5 ± 24.8^a^	44.9 ± 27.7	< 21
Serum total calcium (mmol/l)	2.3 ± 0.1	2.3 ± 0.2	2.2–2.6
Serum ionized calcium (mmol/l)	1.1 ± 0.1	1.2 ± 0.1	1.1–1.3
Urinary calcium (mmol/die)	2.4 ± 1.5	2.8 ± 1.5	2.5–7.5
Parathormone (pmol/l)	5.84 ± 3.60	4.75 ± 2.92	1.05–6.90
25(OH) vitamin D (nmol/l)	35.2 ± 15.0	57.2 ± 25.7	75–125
Serum creatinine (µmol/l)	69.4 ± 18.2	76.8 ± 15.5	44–106
Creatinine clearance (ml/s)	1.5 ± 0.3	1.3 ± 0.4	1.2–2.0
Nadir of ALP (UI/l)	117.0 ± 55.2^a^	66.6 ± 20.3	50–150
Nadir of BAP (mcg/l)	24.7 ± 24.1^a^	8.1 ± 4.1	< 21

Data are presented as mean ± standard deviation or n (percentage). Statistical analysis is performed by Mann–Whitney test and Pearson’s Χ2

*BMI* body mass index, *ALP* alkaline phosphatase, *BAP* bone alkaline phosphatase

^a^*p* < 0.05 vs C-PDB

**Fig. 3:**
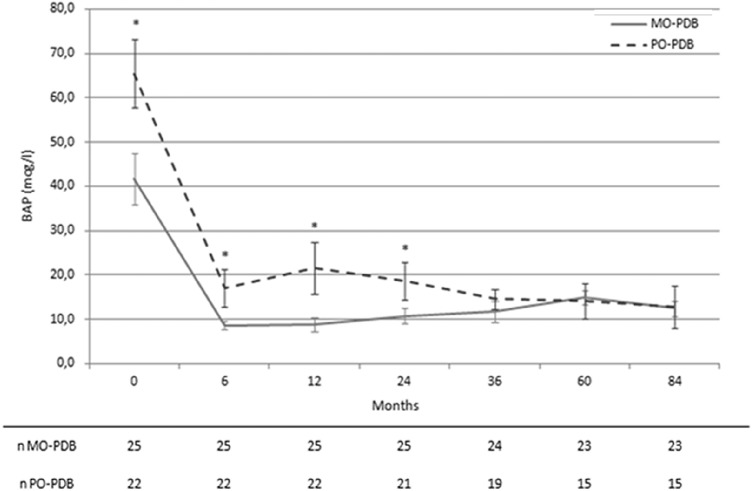
Serum bone alkaline phosphatase (BAP) levels over time in patients with monostotic (MO-PDB) and polyostotic (PO-PDB) Paget’s disease of bone before and after the first infusion of zoledronate. Patients repeating treatment were subsequently excluded from the analysis. Data are represented as mean ± standard error of the mean. *p < 0.05 vs MO-PDB. *n* number of patients assessed at each time point

As shown in Table 2, considering BAP levels after the first zoledronate infusion, PO-PDB showed a lower therapeutic response rate, with a slight higher relapse rate, not reaching statistical significance, in comparison to MO-PDB. Moreover, PO-PDB were more likely to be retreated than MO-PDB, in accordance with a higher nadir of BAP and a longer time to achieve it in the former than in the latter. Moreover, as shown in Fig. 3, after zoledronate infusion, in PO-PDB vs MO-PDB, BAP levels remained higher at 6, 12 and 24 months, while there was no difference at following steps, since retreated patients were subsequently excluded from the analysis.

In models of multivariate logistic regression analysis, after adjusting for age and disease extent, both baseline (Nagelkerke R2 = 0.692; OR 1.09, 95%CI 1.01–1.17) and post-treatment nadir serum BAP (Nagelkerke R2 = 0.541; OR 1.29, 95%CI 1.03–1.62) predicted zoledronate retreatment.

A ROC curve analysis was performed to calculate the cut-off levels of ALP and BAP able to predict the need of retreatment. The optimal threshold values of baseline and post-treatment nadir ALP were 264 UI/l (sensitivity 89%, 95%CI 52–100%; specificity 72%, 53–87%; AUC 0.88, 0.74–0.96) and 98 UI/l (sensitivity 67%, 30–93%; specificity 100%, 90–100%; AUC 0.90, 0.78–0.97), respectively, whereas the baseline and post-treatment nadir BAP values were 75.6 mcg/l (sensitivity 88%, 47–100%; specificity 94%, 80–99%; AUC 0.92, 0.80–0.98) and 9.9 mcg/l (sensitivity 90%, 56–100%; specificity 86%, 70–95%; AUC 0.93, 0.82–0.99), respectively.

## Discussion

Our long-term real-world study show that most PDB patients on intensive zoledronate therapy achieve a clinical and biochemical response at six months, maintaining a good disease control, without relevant disease complications until seven years of follow-up, whereas few patients need retreatment, two years after the first infusion. Moreover, our data display that in comparison to those with persistent disease control, retreated patients are less old and more likely to have PO-PDB, focal spine localizations, pagetic fractures together with higher baseline and post-treatment nadir ALP and BAP levels. After adjusting for confounding factors, we report that baseline and post-treatment nadir BAP levels predict the need of retreatment with a greater accuracy than ALP.

It is well known that for most PDB patients, a single infusion of zoledronate is able to induce a ready response and a sustained remission of the disease [15, 16] through long lasting bone turnover suppression, so it has become the acknowledged first-line drug. Though some experts advise a symptomatic strategy aiming only at bone pain relief [13], a long-term metabolic control through intensive zoledronate therapy is an important goal in the hope of reducing the risk of complications, connoting natural history of PDB [24]. Until now, only two RCTs [12, 13] reported no significant difference in the effect of intensive and symptomatic therapy on clinical outcomes, such as fracture incidence, orthopedic procedures and serious adverse events. However, relevant biases have been reported in these RCTs and future trials aimed at establishing long-term effect of zoledronate therapy on pagetic complications probably will never be performed [24]. Therefore, our long-term observational study could be informative about the incidence of pagetic sequelae, showing only one arthroplasty among disease complications after seven-year follow-up in patients following intensive zoledronate therapy.

Bone turnover markers, including ALP, NTX, PINP and CTX have been used to assess treatment response and relapse [15–19], while scanty data about BAP have been reported until now, though its potential high sensitivity in detecting the activity of PDB [25]. In fact, in our study, in comparison to ALP, BAP is able to detect two more non-responder and a higher number of relapsing patients, whose retreatment potentially prevents pagetic complications. Therefore, we speculate that the greater accuracy of BAP monitoring could overcome its higher cost. However, in our study, the therapeutic response rate according to ALP is similar to that reported by Reid et al. [22] and slight higher than that registered by Devogelar et al. [18] in previous large samples of patients with PDB comparable to ours in terms of age and baseline disease activity. Other studies [17, 26] reported a slight higher rate of non-responder patients after a single infusion of zoledronate, but it has to be noted that they considered small samples of patients. Moreover, in our study, relapse rate according to ALP seems lower than those reported by the Belgian Paget’s Disease Registry [18], but it has to be noted that these Authors considered less stringent criteria to define disease relapse. Furthermore, our results are consistent with those by Reid et al. [15], who found a loss of therapeutic response, corresponding to our definition of relapse, equal to 12.5%. Conversely, other Authors [19] reported a lower relapse rate probably due to (1) its different definition, (2) the enrollment of less severe PDB patients, (3) and, mostly, the use of a different bone turnover marker, i.e. PINP.

On the other hand, poor data about predictors of disease remission and relapse have been reported to date, though their recognition could be useful to identify those few patients needing retreatment. Our data show that patients needing retreatment (1) are less old, (2) they have a greater prevalence of PO-PDB, spine involvement and pagetic fractures, (3) they have higher basal and post-treatment nadir values of both ALP and BAP than subjects achieving and maintaining a good disease control after a single treatment course. In a previous study [15], no difference in age between patients maintaining (70 ± 1 years) and those losing therapeutic response (71 ± 2 years) was found. However, this discrepancy could be due to the lack of heterogeneity in age characterizing their sample, with poor representativeness of less old people. It may be that less old patients, as those included in our study, could have more likely a familial form of PDB, which is characterized by a higher metabolic activity as well as a worse disease control [27]. Moreover, our data suggest for the first time that PO-PDB patients are more likely to have a worse disease control and a high need of retreatment than MO-PDB. Consistently, the greater disease severity of our PO-PDB patients is revealed by higher baseline and follow-up serum BAP levels as well as by a more time to achieve post-treatment nadir values, in comparison to MO-PDB, in line with data by Werner de Castro et al. [28] who reported a correlation between disease extent on bone scintigraphy and baseline serum BAP levels. Even if not previously reported, PO-PDB is reasonably suggestive for a greater burden of disease, due to an accelerated skeletal turnover involving a great amount of bone mass displaying a worse disease control. Similarly, also pagetic fractures could be potential predictors of the need of retreatment, being putative indices of severe PDB, leading to a higher risk of poor response or relapse. Furthermore, among biochemical factors, we find that baseline and post-treatment nadir values of both ALP and BAP are associated with non-responding or relapsing PDB. However, after adjusting for age and disease extent, only BAP levels predict the need of retreatment. In previous study, Patel et al. [20], in sixty patients with PDB following pamidronate therapy, identified pre-treatment and minimum post-treatment ALP levels as significant predictors of the duration of remission. These data are partly consistent with ours, though the two studies differ in criteria to define disease relapse and follow-up period, beyond the administered bisphosphonate. In a more recent study in PDB [15], mean baseline and 6-month bone turnover marker values (particularly ALP, PINP and CTX) after zoledronate infusion were consistently higher in patients losing their therapeutic response. Accordingly, our data show a similar association of ALP levels with disease relapse, but they further demonstrate that BAP could be a better predictor of treatment success. Moreover, as to post-treatment ALP and BAP nadir, our threshold values are in line with recommendations from the Endocrine Society Guidelines on PDB [9], which suggest that a longer duration of remission is associated to bone turnover marker reduction below the midpoint of the reference range. Overall, our data suggest that BAP monitoring is more useful than ALP to identify patients at risk for retreatment, potentially allowing an individualized management in patients with PDB.

On the other hand, we recognize that our study has some limitations. Firstly, its observational retrospective design and the absence of fixed and pre-determined time points, which is however consistent with its real-practice setting. Secondly, the small sample size, that is nevertheless relatively high for a single center, considering the low prevalence of PDB in general population. Moreover, the fact that little more than one third of the subjects has been previously treated with oral bisphosphonates. Furthermore, the lack of information about familial background does not allow us to clarify whether genetic forms could account for the higher need of retreatment observed in less old patients.

In conclusion, our real-world data show that intensive zoledronate therapy is effective in bone pain relief and preventing disease complications through a sustained inhibition of ALP and BAP in most PDB patients. Moreover, after adjusting for clinical confounding factors, both baseline and post-treatment nadir BAP values predict with the greatest accuracy the lack of therapeutic response and disease relapse, i.e. the need of zoledronate retreatment. From a clinical standpoint, this specific bone formation marker could be useful in a tailored management of patients with PDB treated with zoledronate.

## Data Availability

Individual patients data**,** archived in a digital repository, are available on request.
